# Species-Level Analysis of the Human Gut Microbiome Shows Antibiotic Resistance Genes Associated With Colorectal Cancer

**DOI:** 10.3389/fmicb.2021.765291

**Published:** 2021-12-15

**Authors:** Chuanfa Liu, Zhiming Li, Jiahong Ding, Hefu Zhen, Mingyan Fang, Chao Nie

**Affiliations:** ^1^College of Life Sciences, University of Chinese Academy of Sciences, Beijing, China; ^2^BGI-Shenzhen, Shenzhen, China; ^3^China National GeneBank, BGI-Shenzhen, Shenzhen, China

**Keywords:** antibiotic resistance gene (ARG), colorectal cancer (CRC), human gut metagenome, species-level genome bins, *Escherichia coli*

## Abstract

Colorectal cancer (CRC) is the second leading cause of cancer deaths and continuously increases new cancer cases globally. Accumulating evidence links risks of CRC to antibiotic use. Long-term use and abuse of antibiotics increase the resistance of the gut microbiota; however, whether CRC is associated with antibiotic resistance in gut microbiota is still unclear. In this study, we performed a *de novo* assembly to metagenomic sequences in 382 CRC patients and 387 healthy controls to obtain representative species-level genome bins (rSGBs) and plasmids and analyzed the abundance variation of species and antibiotic resistance genes (ARGs). Twenty-five species and 65 ARGs were significantly enriched in the CRC patients, and among these ARGs, 12 were multidrug-resistant genes (MRGs), which mainly included *acrB*, *TolC*, *marA*, *H-NS*, *Escherichia coli acrR* mutation, and *AcrS*. These MRGs could confer resistance to fluoroquinolones, tetracyclines, cephalosporins, and rifamycin antibiotics by antibiotic efflux and inactivation. A classification model was built using the abundance of species and ARGs and achieved areas under the curve of 0.831 and 0.715, respectively. Our investigation has identified the antibiotic resistance types of ARGs and suggested that *E. coli* is the primary antibiotic resistance reservoir of ARGs in CRC patients, providing valuable evidence for selecting appropriate antibiotics in the CRC treatment.

## Introduction

Colorectal cancer (CRC) is one of the most common cancers worldwide and has led to nearly 1 million deaths in 2020 only (Ferlay et al., 2021). Many factors are associated with an elevated risk of CRC, including genetic predisposition, colorectal polyps, inflammatory bowel disease, smoking, and alcohol intake (Wang et al., 2014). Accumulating evidence suggests that long-term, frequent, and/or combined antibiotic use could also be risk factors for CRC (Wang et al., 2014; Dik et al., 2016; Cao et al., 2018; Crockett and Nagtegaal, 2019; Zhang et al., 2019). Antibiotics, such as metronidazole, ciprofloxacin, and rifaximin, are frequently used to treat colitis and intestinal lesions (Bernstein et al., 2016; Nitzan et al., 2016). During the long-term development and progression of CRC, the detrimental effect of antibiotics may be present even at the early stage of colitis, adenomatous polyps, or other precursors of the CRC. It is worth noting that antibiotic use could increase the richness of antibiotic-resistance bacterial species and the abundance of antibiotic resistance genes (ARGs) in the gut microbiota (Casals-Pascual et al., 2018; Dubinsky et al., 2020). Subsequent to antibiotic use and increased resistance, bowel dysbacteria may occur, and concomitantly, colonization resistance, and mucus production of the colon mucosal may be impaired (Becattini et al., 2016; Schwartz et al., 2020). Existing literature indicates that gut microbiota dysbiosis and colon mucosal surface changes are associated with the occurrence and progression of CRC (Yachida et al., 2019; Cheng et al., 2020; Xing et al., 2021). Therefore, research on drug-resistant microbiota and resistance genes may help to understand the progression of CRC.

The compositional patterns of antibiotic-resistant species and ARGs in the gut microbiota of CRC patients were scantly studied. To examine their potential effects exerted upon CRC patients and healthy people, we have downloaded published human gut metagenomic data of CRC patients and healthy controls to study the antibiotic resistance species and ARG distribution in the gut microbiota. We performed the metagenomic assembly to obtain representative species-level genome bins (rSGBs) to investigate ARG abundance in each species. Based on the Genome Taxonomy Database (GTDB) and Comprehensive Antibiotic Resistance Database (CARD), we annotated species and ARGs in rSGBs and analyzed their abundance. These analyses have revealed how the burden of antibiotic resistance changes in the intestine of CRC patients, stressing significant associations between these changes and microbiota composition. Our study has characterized the resistance of the gut microbiota in CRC patients and may shed new light on the proper antibiotic use for avoiding drug resistance.

## Results

### Reconstruction and Annotation of Microbial Genomes and Plasmids

In this study, we downloaded metagenomic data of 382 CRC patients (the CRC group) and 387 healthy controls (CTR group) from eight studies (Table 1 and Supplementary Table 1). The genome reconstruction was performed using a pipeline reported by Pasolli et al. (2019) and carried out a *de novo* single-sample metagenomes assembly and binning. More than 23.5 million contigs (mean ± SD, 30,688.6 ± 13,972.6) were assembled from these samples; 5,880 high-quality metagenome-assembled genomes (MAGs) and 5,390 medium-quality MAGs were obtained (Supplementary Table 2). After clustering and filtering the rSGBs for the high-quality MAGs, we obtained 696 rSGBs with genome sizes ranging from 0.95 to 6.41 Mb (2.39 ± 0.43 Mb) (Supplementary Tables 2–4). We then aligned the high-quality sequencing reads to the 696 rSGBs. The read mapping rate in our results (76.6% ± 7.8%, Supplementary Table 2) was similar to that of a large-scale gut microbiota study (range, 67.76–87.51%) (Pasolli et al., 2019). Based on the quality of the mapping rate, it is acceptable to use our data for subsequent species and ARG annotations.

**TABLE 1:** Characteristics of the data sets included in this study.

Accession number^a^	Group (n^b^)	Age (years) (mean ± SD^c^)	Gender (F/M,%^d^)	BMI (kg/m^2^) (mean ± SD^c^)	Read counts (mean ± SD^c^)
PRJEB7774 Feng et al. (2015)	CRC (46)	67.07 ± 10.91	39.13/60.87	26.46 ± 3.54	49,936,552 ± 7,270,051
	CTR (63)	67.06 ± 6.37	41.27/58.73	27.57 ± 3.74	46,090,975 ± 7,068,141
PRJNA389927 Hannigan et al. (2018)	CRC (28)	58.86 ± 11.02	28.57/71.43	28.57/71.43	4,851,235 ± 2,209,656
	CTR (28)	55.46 ± 9.52	60.71/39.29	60.71/39.29	5,848,176 ± 3,578,299
PRJEB10878 Yu et al. (2017)	CRC (74)	66.04 ± 10.60	35.14/64.86	23.98 ± 3.16	49,225,941 ± 10,554,038
	CTR (54)	61.76 ± 5.67	38.89/61.11	23.46 ± 2.96	53,622,545 ± 8,128,850
PRJEB6070 Zeller et al. (2014)	CRC (91)	64.66 ± 12.23	40.66/59.34	26.04 ± 4.49	43,899,076 ± 19,556,571
	CTR (66)	58.61 ± 12.79	50.00/50.00	24.68 ± 3.17	48,152,424 ± 23,181,408
PRJEB27928 Wirbel et al. (2019)	CRC (22)	66.55 ± 10.6	50.00/50.00	25.33 ± 4.93	48,769,786 ± 18,597,297
	CTR (60)	57.57 ± 11.08	46.67/53.33	24.88 ± 3.2	28,494,527 ± 7,144,313
PRJNA447983 Thomas et al. (2019)	CRC (29)	71.45 ± 8.23	20.69/79.31	25.71 ± 4.14	46,797,011 ± 22,256,056
	CTR (24)	67.92 ± 7.01	45.83/54.17	25.32 ± 3.51	58,117,998 ± 40,294,533
PRJDB4176 Yachida et al. (2019)	CRC (40)	59.05 ± 12.83	47.50/52.50	22.36 ± 2.72	40,249,523 ± 12,291,484
	CTR (40)	63.63 ± 12.36	42.50/57.50	22.90 ± 2.44	46,232,480 ± 14,228,285
PRJEB12449 Vogtmann et al. (2016)	CRC (52)	61.85 ± 13.58	28.85/71.15	24.89 ± 4.25	52,664,424 ± 16,619,909
	CTR (52)	61.23 ± 11.03	28.85/71.15	25.34 ± 4.28	52,629,971 ± 12,022,921

*^a^References of the study.*

*^b^Counts of samples.*

*^c^Standard deviation.*

*^d^Ratio of the percentage of female and male.*

**FIGURE 1:**
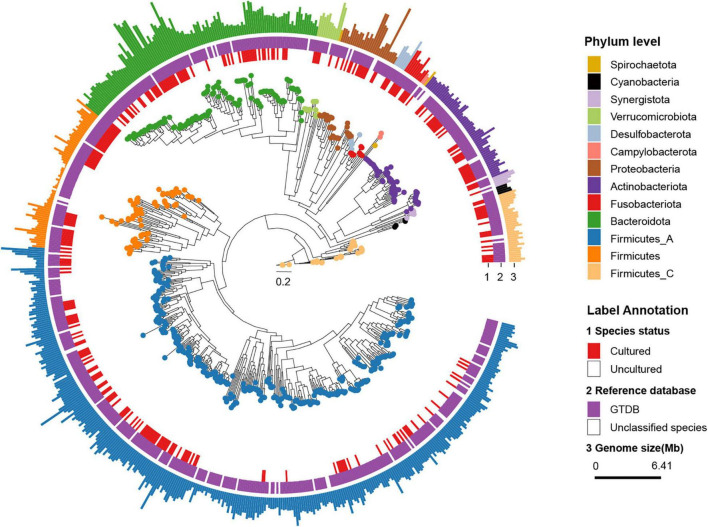
Phylogenetic tree of representative genomes. The phylogenetic tree in the center showed 696 rSGBs. The colored points on the tree represent different phyla. The innermost circle (labeled “1”) means whether the species was cultured (red color, marked as Cultured) or not (white color, marked as Uncultured) in the previous study. The middle circle (labeled “2”) means whether the species could be annotated to the known genome (purple color, marked as “GTDB”) or not (white color, marked as “Unclassified species”) in the GTDB. In the outmost circle (label “3”), the length of bars represents the genome size (bp), and colors represent different phyla.

**FIGURE 2:**
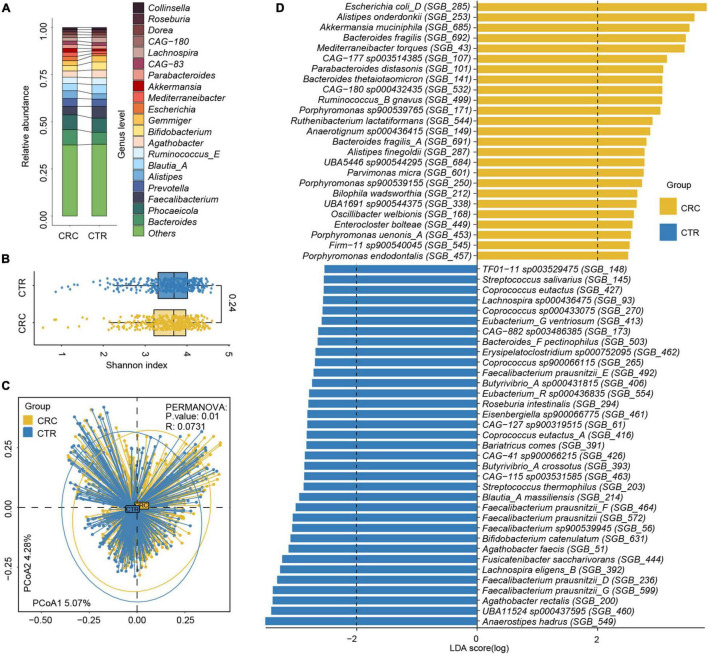
Relative abundance difference of rSGBs in the CRC and CTR groups. **(A)** The relative abundance of rSGBs on the genus level. **(B)** The Shannon–Wiener indices of rSGBs abundance in CRC (yellow color) and CTR (blue color) groups were similar (Wilcoxon rank-sum test, *p* = 0.24). **(C)** Principal coordinate analysis (PCoA) plot depicted the Bray–Curtis distances of rSGBs in the CRC (yellow) and CTR (blue) groups. *P*-value and R represent PERMANOVA analysis *p*-value and *R*^2^ value, respectively. **(D)** LefSe analysis results showed the distribution difference of rSGBs, the colors yellow and blue represent the CRC and CTR groups, respectively.

Thereafter, we classified rSGBs using the GTDB Toolkit (GTDB-Tk, see *Methods* for details on the taxonomy nomenclature used) (Chaumeil et al., 2019). However, 60 rSGBs (8.62%) could not be assigned to an existing species, and 402 (57.75%) rSGBs belonged to uncultured species (Figure 1 and Supplementary Table 5). All 696 rSGBs were classified into 13 phyla and 306 genera. In line with previous studies (Yeoh et al., 2020), Firmicutes (including Firmicutes A), Bacteroidota, Actinobacteriota, and Proteobacteria were predominant phyla, and the total relative abundance accounted for more than 90% of the gut microbiota (mean ± SD, CRC group: 93.69% ± 8.76%; CTR group: 96.67% ± 5.75%) (Supplementary Figure 1A). *Bacteroides*, *Phocaeicola*, *Faecalibacterium*, *Prevotella*, *Alistipes*, and *Blautia A* were the dominant genera in the gut (Figure 2A). It indicated that our rSGBs covered the dominate species in the gut microbiota.

In addition, we assembled the plasmids of gut microbiota using metaplasmidSPAdes (Antipov et al., 2019). We obtained 24,692 plasmid-sourced contigs (N50 = 42,448 bp; max = 473,623 bp; min = 2,564 bp) with a mean of 32 contigs in each sample. The plasmid-sourced genes were predicted and clustered with MetaGeneMark and cd-hit, respectively (Li and Godzik, 2006; Zhu et al., 2010). A non-redundant plasmid-sourced gene catalog (159,890 genes, N50 = 701 bp) was obtained. Next, we applied reference-based taxonomy annotation of the gene catalog using the NCBI-NT database. Finally, 157,504 (98.5%) of the genes in the gene catalog could be uniquely and reliably assigned to a species. We found those genes mainly from *Escherichia coli*, *Faecalibacterium prausnitzii*, *Bacteroides dorei*, *Bacteroides fragilis*, *Bacteroides uniformis*, and *Klebsiella pneumoniae*.

### Alterations of Gut Microbial Composition in Colorectal Cancer and CTR Groups

We analyzed the effect size of cohorts and host characteristics on the variance of the gut microbiome by permutational multivariate analysis of variance (PERMANOVA) test, where results revealed the factor “cohort” to have a predominant impact on the species and ARG composition of the subjects (Supplementary Figure 2). To test the accuracy of results in the analysis, we select two cohorts randomly to confirm our species and ARG results [PRJEB7774 (*n* = 109) and PRJEB12449 (*n* = 104)]. The analysis of the α and β diversity of microbial composition revealed that the CRC group had a slightly lower species diversity than the CTR group [Shannon–Wiener index (*H’*), *p* = 0.24, Figure 2B], which was consistent with the literature (Gao et al., 2015). The dimensionality-reduction analysis [principal coordinate analysis (PCoA) and non-metric multidimensional scaling (NMDS) analysis] of the rSGBs relative abundance of the CRC and CTR groups showed that the CRC and CTR groups were separated (PERMANOVA analysis *p* = 0.01, *R* = 0.0731) (Figure 2C and Supplementary Figure 1B).

Then, the composition of microbiota between CRC patients and healthy controls was compared at phylum and genus levels. On the phylum levels, Bacteroidota, Desulfobacterota, and Fusobacteriota phyla were enriched in the CRC group, and Firmicutes A phylum was enriched in the CTR group (Wilcoxon test, adjusted *p* < 0.05; Supplementary Figure 1C). On the genus level, *Anaerotignum*, *Bilophila*, *Bulleidia*, *Flavonifractor*, *Gemella*, *Intestinimonas*, *Parvimonas*, *Peptostreptococcus*, *Porphyromonas*, *Prevotella*, and *Ruthenibacterium* genera were enriched in the CRC group; meanwhile, *Agathobacter*, *Anaerostipes*, *Butyricicoccus A*, *Butyrivibrio A*, *CAG-41*, *Eubacterium G*, *Eubacterium R*, *Faecalibacterium*, *GCA-900066135*, *Lachnospira*, *TF01-11*, and *UBA11524* genera were enriched in the CTR group significantly (Wilcoxon test, adjusted *p* < 0.05; Supplementary Figure 1C).

Next, we compared the microbiota composition between CRC patients and healthy controls at species levels using the linear discriminant analysis effect size (LefSe) algorithm (Segata et al., 2011). Within the 25 species enriched in the CRC group (Figure 2D and Supplementary Table 6), nine species had been reported to be increased in the CRC group before, that is, *E. coli* (GTDB classification: *E. coli D*), *Parabacteroides distasonis*, *B. fragilis*, *Porphyromonas* species, *Alistipes finegoldii*, *Alistipes onderdonkii*, *Akkermansia muciniphila*, *Bacteroides thetaiotaomicron*, *Mediterraneibacter torques* (previously named *Ruminococcus torques*), and *Ruminococcus B gnavus* (Zhang et al., 2018; Ai et al., 2019; Dai et al., 2019; Sahankumari et al., 2019; Wong and Yu, 2019; Yang et al., 2019). Moreover, two species were the first discovered species that were enriched in the CRC group, that is, *CAG-180 sp000432435* and *CAG-177 sp003514385*. Among the 35 species enriched in the CTR group (Figure 2D and Supplementary Table 6), *Anaerostipes hadrus*, *Bifidobacterium catenulatum*, *Fusicatenibacter saccharivorans*, and butyrate-producing species *F. prausnitzii*, *Agathobacter rectalis*, and *Agathobacter faecis* had been reported in the literature as enriched in the healthy controls and possibly beneficial to the gut health (Ai et al., 2019; Kim et al., 2020; Ma et al., 2021). Focusing on the 60 microbiota that exhibited significantly different abundances between the CRC and CTR groups in all samples, we further compared the relative abundances of these microbiota between the CRC and CTR groups in PRJEB7774 and PRJEB12449 cohorts using the Wilcoxon rank-sum tests. We found that the enrichment of 85 and 83.3% of the significantly different species (CRC and CTR) in the PRJEB7774 and PRJEB12449 cohorts were congruent with those in the composite cohort of all samples (Supplementary Figure 3).

### Antibiotic Resistance Genes Conferred to Multiple Antibiotics

**FIGURE 3:**
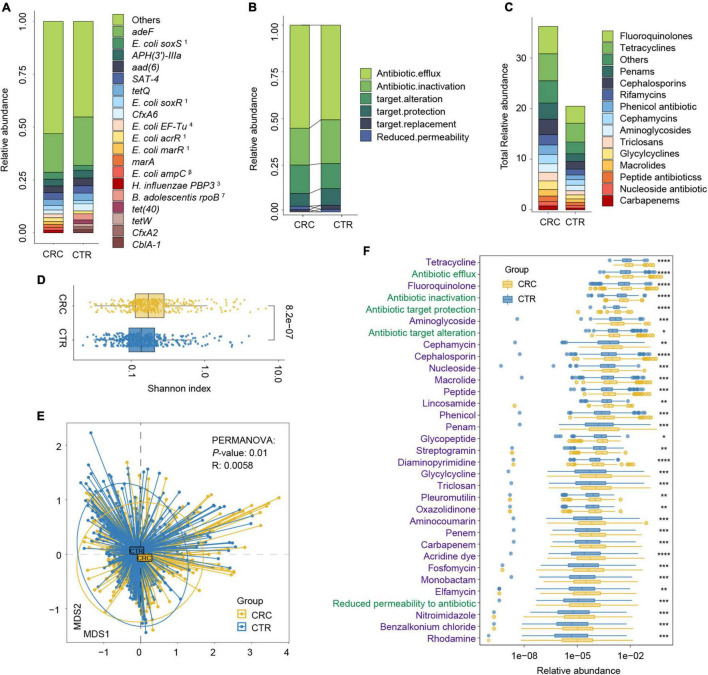
Antibiotic resistance variations in CRC and CTR groups. **(A)** The average abundance percentage of ARGs in the CRC and CTR groups. **(B)** Resistance mechanism abundance percentage in the CRC and CTR groups, respectively. **(C)** The abundance of resistance drug types in CRC and CTR groups. **(D)** Shannon–Wiener index (*H’*) difference of ARG abundance in the CRC (yellow color) and CTR (blue color) group. **(E)** NMDS results of ARG abundance in the CRC (yellow color) and CTR (blue color) group. **(F)** Resistance drug types (green color label) and resistance mechanisms (purple color label) abundance had statistical differences in the CRC (yellow color) and CTR (blue color) groups. The following symbols denoted statistical significance: *p < 0.05, **p < 0.01, ***p < 0.001, ****p < 0.0001. In **(A)**, the superscript labels from numbers 1 to 7 and β represent the resistance drug types of mutations: ^1^antibiotic resistance, ^2^ multiple antibiotics, ^3^β-lactam antibiotics, ^4^pulvomycins, ^5^fosfomycin antibiotics, ^6^fluoroquinolones, ^7^rifampicins; ^β^ β-lactamases.

To analyze the antibiotic resistance information in microbiota, we characterized the ARGs and analyzed their abundance in the rSGBs and plasmids by annotating them to the CARD (Alcock et al., 2020), obtaining 164 ARGs in 189 rSGBs (Supplementary Tables 7, 8). The top 11 ARGs accounted for 51.16% of all abundance, mainly including *adeF*, *TolC*, *E. coli soxS* mutation, *AcrS*, *E. coli soxR* mutation, and *marA* (Figure 3A and Supplementary Figure 4A). The *adeF* had the highest relative abundance, which could encode the membrane fusion proteins of the multidrug efflux complex AdeFGH (Coyne et al., 2010). Within the plasmid-sourced genes, we obtained 43 ARGs in 49 genes (0.03% of all plasmid genes) that conferred resistance to antibiotics (Supplementary Table 8), and the ARG *tetQ* had the highest abundance.

We analyzed the resistance mechanisms and resistance drug types of identified ARGs and found that these ARGs in the rSGBs and plasmids could confer resistance to 33 and 18 types of antibiotics, respectively. Notably, 53.05% of rSGB-sourced ARGs (87 out of 164) could confer resistance to more than one antibiotic (Supplementary Table 7). ARGs could affect antibiotic resistance through the mechanisms of antibiotic efflux, inactivation, target alteration, target protection, target replacement, and reduced permeability to antibiotics.

Regarding the percentage of abundance, the antibiotic efflux accounted for 53.36% of rSGB-sourced ARGs resistance mechanism, and antibiotic inactivation and antibiotic targets alteration accounted for 21.21 and 14.49%, respectively (Figure 3B and Supplementary Figure 4B). Eighty percent of the total rSGB-sourced ARG abundance could be ascribed to the top 10 resistance drug types, including nucleoside antibiotics (17.3%), cephalosporins (16.77%), macrolides (11.64%), phenicol antibiotics (8.06%), fluoroquinolones (5.97%), penams (5.2%), rifamycins (2.96%), and other antibiotics (Figure 3C and Supplementary Figure 4C).

We found that those with top resistance types were also the most consumed antibiotics globally (Van Boeckel et al., 2014). For example, ARGs in the gut of the CRC group could confer six types of global high-consumption antibiotics, including penicillins, cephalosporins, macrolides, fluoroquinolones, tetracyclines, and rifamycins. The antibiotic resistance types in our findings were consistent with the global antibiotic consumption. Among these antibiotics, 19.23% belong to the Access Class, 30.77% belong to the Watch Class, and 23.08% are part of the Reserve Class (World Health Organization AWaRe classification, version 2019; Supplementary Table 9 and Supplementary Figure 4D; World Health Organization, 2019).

### The Divergence and Heterogeneity of Antibiotic Resistance Gene in the Colorectal Cancer and CTR Groups

We performed a group comparison in the abundance of ARGs, antibiotic mechanisms, and their resistance drug types to analyze ARG variations. First, the component of ARGs in the CRC and CTR groups were analyzed. Compared with the CTR group, the CRC group had a higher rSGB-sourced ARG diversity [Shannon–Wiener index (*H’*), *p* < 0.001, Figure 3D], which indicated that gut microbiota in the CRC group had more complexity of ARGs. The β diversity of ARG abundance showed that the ARGs in the CRC group differed from those in the CTR group (NMDS, PERMANOVA: *p* = 0.01, *R* = 0.0058) (Figure 3E).

We then analyzed the rSGB-sourced ARG abundance grouped by resistance mechanisms and resistance drug types in the CRC and CTR groups. Eighty percent (33 of 39) resistance mechanisms and resistance drugs were significantly enriched in the CRC group, mainly including antibiotic efflux and inactivation mechanism, tetracyclines, fluoroquinolones, penams, carbapenem, and cephalosporins (Figure 3F). The abundance of ARGs in the rSGBs was significantly enriched in the CRC group on the mechanisms and resistance drug type scales.

**FIGURE 4:**
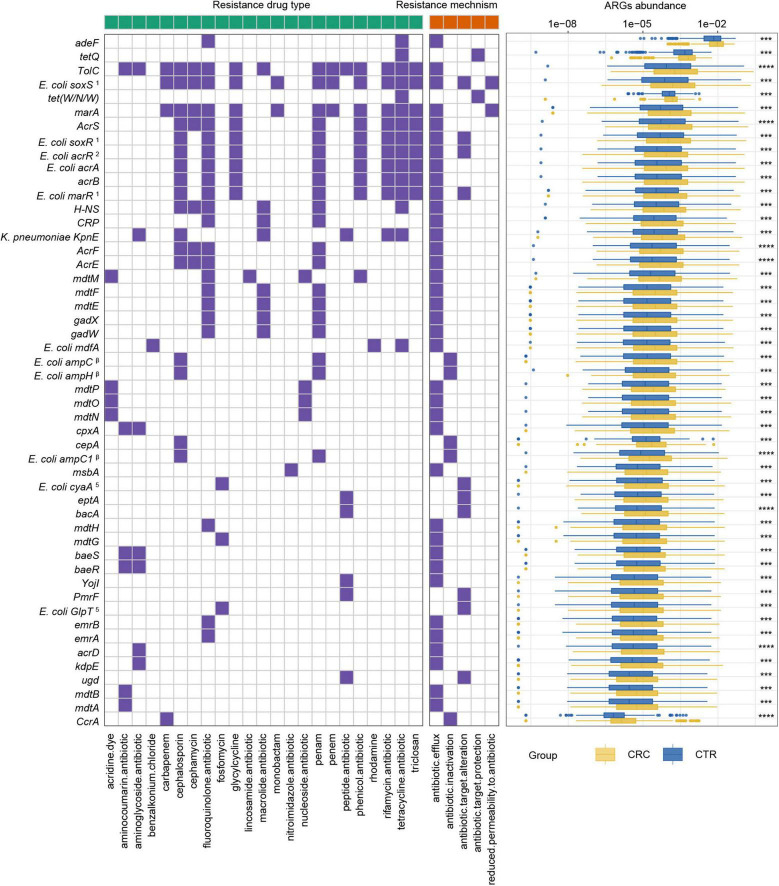
ARGs with significant differences and their resistance mechanism and resistance drug type abundance. The left (with a green label) and the right (with an orange label) panels of heatmap represented drug resistance type and resistance mechanism, respectively. The purple or white color denoted with and without certain characteristic. The boxplot showed abundance difference of ARGs in the CRC (yellow) and CTR (blue) groups. The following symbols denoted statistical significance: ***p < 0.001, ****p < 0.0001. The superscript label from numbers 1 to 7 and β was the same as Figure 3A.

**FIGURE 5:**
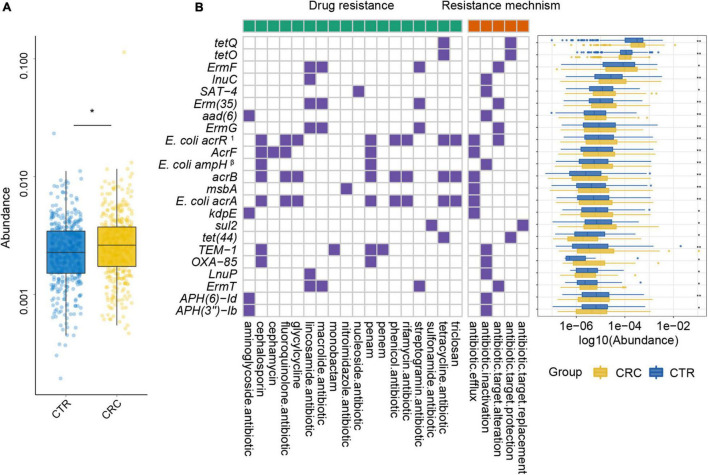
Plasmid ARGs with significant differences and their resistance mechanism and resistance drug type abundance. **(A)** The abundance variation of total ARGs from plasmids in the CRC (yellow color) and CTR (blue color) groups. **(B)** The left (with a green label) and the right (with an orange label) panels of the heatmap represented drug resistance type and resistance mechanism, respectively. The purple or white color denoted with or without certain characteristic. The boxplot showed abundance difference of ARGs in the CRC (yellow color) and CTR (blue color) groups. In **(B)**, the superscript label 1 and β represent the resistance drug types of mutations: ^1^multiple antibiotics; ^β^ β-lactamases. The following symbols denoted statistical significance: *p < 0.05, **p < 0.01.

To further demonstrate the variability in the resistance burden between the CRC and CTR groups, we analyzed the abundance difference of every ARG. Fifty ARGs from rSGBs were significantly enriched in the CRC group (Wilcoxon test, adjusted *p* < 0.05) (Figure 4). Correspondingly, the total abundance of ARGs from plasmids was higher in the CRC group than that in the CTR group (Figure 5A), and 70% of ARGs (16 of 23) were enriched in the CRC group significantly (Wilcoxon test, adjusted *p* < 0.05) (Figure 5B). In our two validation cohorts, 100 and 96% rSGB-sourced ARGs as well as 60.9 and 73.9% plasmid-sourced ARGs in PRJEB7774 and PRJEB12449 cohorts were enriched congruently with all the samples (Supplementary Figures 5, 6). The ARGs from both the rSGBs and plasmids in the validation cohorts showed a notable consistency with those in all samples.

Among these rSGB-sourced ARGs, 90% (45 of 50) were found in the *E. coli*, mainly composing of *TolC*, *E. coli soxS* mutation, *marA*, *AcrS*, *acrB*, and *mdtM*. The remaining five ARGs, including *adeF*, *CcrA*, *cepA*, *tet(W/N/W)*, and *tetQ*, were found in *P. distasonis, B. fragilis A*, *B. fragilis*, *UMGS693 sp900544555*, and *A. onderdonkii*, respectively. Although the ARGs existing in *E. coli*, *Bacteroides* species, and *Alistipes* species had been reported, their association with CRC was not robust (Dubinsky et al., 2020; Parker et al., 2020). In terms of resistance mechanisms and resistance drug types, these rSGB-sourced ARGs enriched in CRC could confer resistance to 33 types of antibiotics by five mechanisms, and 37 of 50 ARGs encoded antibiotic efflux mechanism (Figure 4). In addition, in rSGB-sourced ARGs, 22 ARGs had resistance to fluoroquinolones, 20 ARGs to penams, 17 ARGs to cephalosporins, 15 ARGs to tetracyclines, and 10 ARGs to rifamycins, respectively.

For the 23 plasmid-sourced ARGs, 39% of ARGs (9 of 23) were found in *E. coli*, mainly composing *acrB*, *msbA*, *AcrF*, and *E. coli acrR* mutation. These ARGs could confer resistance to 18 antibiotics by five mechanisms (Figure 5B), where the antibiotics included cephalosporins, fluoroquinolones, macrolides, tetracycline, and penams. Seven ARGs had resistance to penams and cephalosporins, six ARGs to tetracyclines and lincosamides, respectively.

Interestingly, penicillins, cephalosporins, and fluoroquinolones had been reported to be associated with the onset of colitis (Nitzan et al., 2016). Meanwhile, fluoroquinolones, cephalosporins, tetracyclines, and rifamycins were the most commonly used antibiotics in colitis treatment (Theochari et al., 2018). These results suggested that the increase in resistance burden might be due to antibiotic treatment of precancerous colitis and antibiotic use related to intestinal colitis and CRC by an unknown mechanism.

**FIGURE 6:**
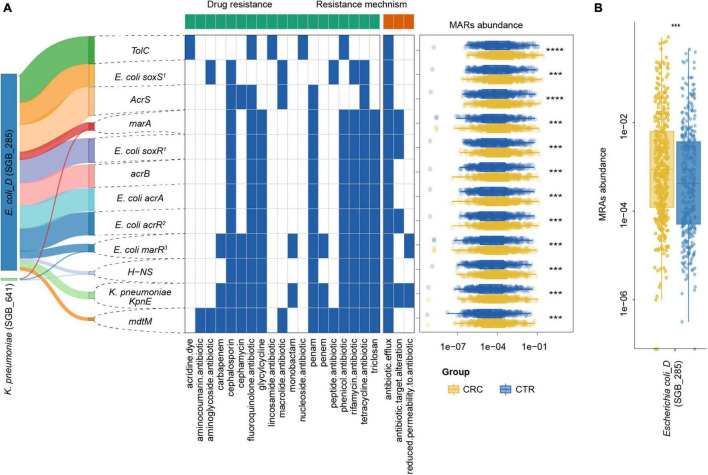
MRG distribution in the CRC and CTR groups. **(A)** Distribution, resistance drug types, and resistance mechanism of MRGs in CRC (yellow color) and CTR (blue color) groups. The left Sankey showed the source percentage of MARs. The middle heatmap exhibited the drug resistance type and mechanism of MARs. The blue color denoted MAR had that characteristic. The right boxplot showed the abundance of MARs. **(B)** MRGs in *Escherichia coli* (SGB_285) were enriched in the CRC groups significantly. The following symbols denoted statistical significance: ***p < 0.001, ****p < 0.0001.

To further investigate the multidrug resistance of ARGs, we denoted ARGs that could confer resistance to five or more antibiotic drug types as multidrug-resistant genes (MRGs) (Supplementary Table 7). A total of 21 species from rSGBs were found that carried 41 MRGs, of which 12 MRGs were significantly enriched in the CRC group (Supplementary Figure 7). In the plasmid-sourced ARGs, three MRGs were found (*acrB*, *E. coli acrR* mutation, and *E. coli acrA*) enriched in the CRC group. All these MRGs enriched in the CRC group were derived from *E. coli* and *K. pneumoniae* (Figures 5B, 6A). Meanwhile, compared with the CTR group, the rSGB-sourced MRG abundance in *E. coli* was significantly higher in the CRC group (Figure 6B). These MRGs could confer resistance to 19 types of antibiotic drugs, which included fluoroquinolones, cephalosporins, tetracyclines, penams, phenicol antibiotics, and rifamycins (Figures 5B, 6A). Among these MRGs, *mdtM* could confer resistance to 15 types of antibiotics, where both *E. coli marR* mutant and *K. pneumoniae KpnE* could confer resistance to 12 types of antibiotics, and *H-NS* conferred resistance to 9 types of antibiotics (Figure 6A). The intergroup difference of MAGs was completely confirmed in the PRJEB7774 and PRJEB12449 cohorts, respectively (Supplementary Figure 8). This finding showed that the abundance of MRGs and species with MARs was enriched in the CRC group, which suggested that the increase in MRG abundance was also associated with the CRC. Findings thus far indicated that the CRC group had a higher antibiotic resistance burden and had resistance to multiple antibiotics.

### Species Encoded by Antibiotic Resistance Genes Were Enriched in the Colorectal Cancer Group

**FIGURE 7:**
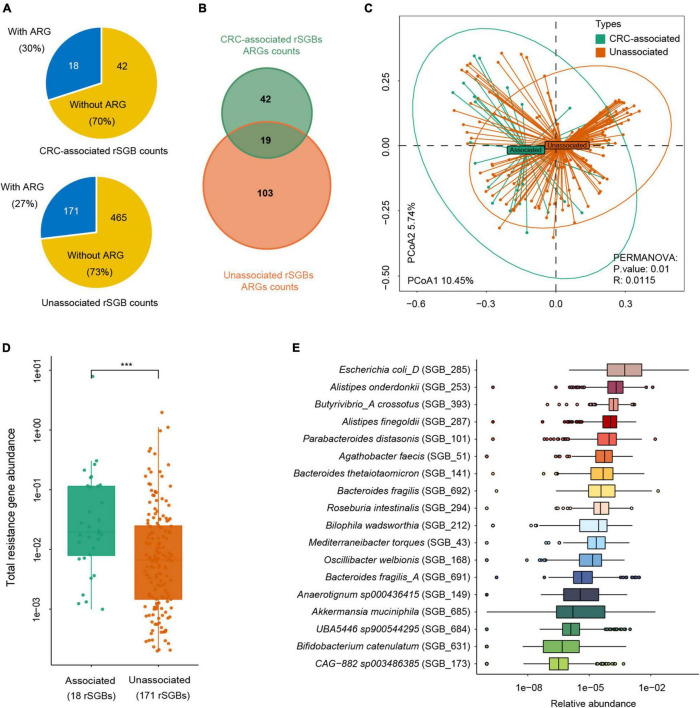
Abundance difference of ARGs in rSGBs that associated with CRC or not. **(A)** The rSGB count and percentage in the CRC-associated and unassociated clusters; the blue or yellow colors represented the proportion of rSGBs with or without ARGs. **(B)** ARG counts in the CRC-associated and unassociated clusters. **(C)** PCoA plot depicting Bray–Curtis distances of ARG abundances in the CRC-associated (green color) and unassociated (dark yellow color) clusters. **(D)** Total ARG abundance in the CRC-associated (green color) and unassociated (dark yellow color) clusters (***p < 0.001). **(E)** Relative abundance of ARGs in every CRC-associated rSGBs in the CRC groups.

We further analyzed the sum of ARG abundance in the CRC-associated and unassociated species, where species enriched in the CRC and CTR groups were denoted as the “CRC-associated” cluster and others as the “unassociated” cluster. In the CRC-associated cluster, there were 30% of species (18 of 60 species) carrying ARGs; accordingly, there were 27% of species (171 of 636 rSGBs) with ARGs in the unassociated cluster (Figure 7A). Among all ARG types, 61 ARG types are found in CRC-associated clusters, and 122 ARG types are in unassociated clusters, whereas 19 are common to both clusters (Figure 7B). Moreover, the results showed that the CRC-associated cluster and unassociated cluster were divided in the PCoA analysis (PERMANOVA analysis, *p* = 0.01, Figure 7C), and the total abundance of ARGs in the CRC-associated cluster was significantly higher than that in the unassociated cluster (Wilcoxon test, *p* < 0.0001, Figure 7D).

The analysis of ARG abundance in the CRC group reported that *E. coli*, *A. onderdonkii*, *P. distasonis*, and *A. finegoldii* had a relatively high abundance of ARGs (median value) (Figure 7E). Notably, these four species were also significantly enriched in the CRC groups, primarily *E. coli*, which was encoded by as many as 37 ARGs and 13 MRGs. Although the ARG type counts in the CRC-associated cluster were lower than that in the unassociated cluster, the former cluster carried a higher abundance of ARGs. Therefore, *E. coli* in the CRC-associated cluster may act as an antibiotic resistance reservoir in the gut microbiota because of its high abundance of drug resistance genes. Taken together, we reported here that more ARGs encoded CRC-associated species than unassociated species, and *E. coli* was a critical antibiotic reservoir in the gut.

### Predictive Effectiveness of Species and Antibiotic Resistance Genes

To demonstrate the plausible clinical prediction of ARGs and species, we next built a series of random-forest prediction models using all the species, rSGB-sourced ARGs, and these features enriched in two groups. First, we tested the classification effect of the abundance of species and ARGs. The results showed that the classification performance of species features (area under the curve [AUC] = 0.802) outperformed that of the ARG features (AUC = 0.663) (Supplementary Figures 9A,B).

**FIGURE 8:**
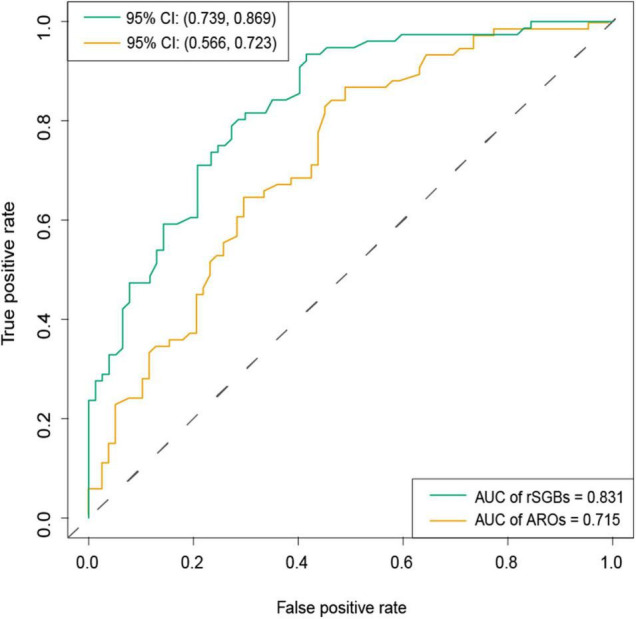
AUC of classification model by selected rSGBs and ARGs features. The random forest model was built based on species (green color) and ARGs (dark yellow). CI, confidential interval.

To improve the precision, we rebuilt two models using 118 selected species (32 carried ARGs) features and 19 selected ARG features, using the random forest method (Supplementary Table 10 and Supplementary Figures 9C,D). From Figure 8, it could be observed that the species model was more effective (AUC = 0.831) than the ARGs model (AUC = 0.715), when classifying the CRC and CTR groups. Our species-based classification model performed better than the previous report, where the AUC ≥ 0.8 (Wirbel et al., 2019). The model accuracy based on ARG features in our study was approximate to that of a previously reported species model, although the accuracy of our model was unsatisfying (Thomas et al., 2019). In summary, species and ARGs in microbiota could predict CRC patients with modest precision.

## Discussion

Antibiotic resistance is one of the most critical public health threats to human beings in recent years (Hernando-Amado et al., 2019). Although evidence has shown that antibiotic use may increase the risk of CRC, whether antibiotic resistance is also related is far from clear (Dik et al., 2016; Zhang et al., 2019; Armstrong et al., 2020; Wan et al., 2020). Here, we discovered 696 rSGBs, 24,692 plasmids, and 187 ARGs in the gut microbiota, where 25 species were enriched in the CRC group, and 13 species carried ARGs, such as *E. coli*, *A. onderdonkii*, *B. fragilis*, *A. muciniphila*, and *M. torques*. The abundance of ARGs was enriched in the CRC group, and *E. coli* was the essential ARG carrier.

To analyze the ARGs on the species level, we reconstructed the genomes *via* single-sample assembly from the metagenomes as reported in the literature (Pasolli et al., 2019; Zhu et al., 2019). However, plasmids also harbor many ARGs. It is reported that while the bacterial genome is assembled, its plasmids often remain unidentified because it is not clear which contigs in the genome assembly have arisen from plasmids (Antipov et al., 2016, 2019). We used metaplasmidSPAdes, which could identify novel plasmids and assemble plasmids from metagenomic data sets in order to annotate as many ARGs as possible.

With the species-level genomes reconstructed, representative genomes had a high genome quality of more than 90% completeness and less than 5% contamination. As a result, we found 25 species enriched in the CRC group, such as *E. coli*, *P. distasonis*, *A. muciniphila*, *B. thetaiotaomicron*, etc. These species had been reported to induce CRC by producing inflammatory polysaccharides, cell cycle inhibiting factors, and cytolethal distending toxins (Wassenaar, 2018; Sahankumari et al., 2019). For species enriched in the CTR group, mounting evidence showed the potential benefits of *F. prausnitzii* for improving intestinal healthy *via* producing butyrate (Ferreira-Halder et al., 2017; Kim et al., 2020). *B. catenulatum*, as a significant commensal bacterium of *F. prausnitzii*, could improve its growth, gut colonization, and butyrate production by producing short-chain fatty acids (Kim et al., 2020). Moreover, *Lachnospira eligens* (formerly *Eubacterium eligens*) could effectively suppress intestinal inflammation and prevent colitis and CRC (Feng et al., 2016). Although more than 90% of species we found had been reported or cultured before, we had also discovered unknown species enriched in the CRC group, such as *UBA5446 sp900544295* and *Anaerotignum sp000436415*, with both of the species carrying ARGs. It indicated that reconstructing genomes was useful to find out more species carrying ARGs and increased the probability of illustrating the ARG distribution in gut microbiota.

In these species of rSGBs, we detected 164 ARGs, which was slightly higher than another large cohort study on antibiotics (149 ARG types) (Hu et al., 2013). In our results, 30.49% of ARGs were enriched in the CRC group significantly. These CRC-associated species had a high antibiotic resistance abundance. The *adeF* gene was the largest abundant ARG in the CRC group. In another cohort of a healthy population, the highest abundance of ARG was *TcR* (Hu et al., 2013). The ARG abundance difference in different populations may be due to the disease-specific variations in antibiotic resistance under different antibiotic exposures (Shamsaddini et al., 2021).

Antibiotic resistance could be acquired *via* gut microbiota through the use of broad-spectrum antibiotics, including cephalosporins, fluoroquinolones, penams, and rifaximin, to name a few. We noticed that the nucleoside antibiotic type had the highest abundance of antibiotics, followed by the cephalosporins, macrolides, and phenicol antibiotic types. Although a previous meta-analysis reported no significant associations between CRC and some antibiotics, for example, quinolones, tetracyclines, and macrolide antibiotics (Wan et al., 2020), a significantly higher abundance of these antibiotic resistance drugs was detected in the CRC group. In this study, penams were significantly higher in the CRC group, which may be caused by higher penicillin usage in CRC patients (Wan et al., 2020). Another interesting finding was that we found no evident difference in resistance to rifaximin between the CRC and CTR groups. Rifaximin, a rifamycin antibiotic, was popularly used in travelers’ diarrhea and irritable bowel syndrome. It had been reported to neither affect ARGs nor increase the ARG burden because the use of rifaximin would rarely bring about the development of drug resistance compared with other antibiotics (Shamsaddini et al., 2021). The high ARG burden in CRC patients suggested that it is recommended to consider possible antibiotic resistance when selecting appropriate antibiotic treatments, such as short-term alternating antibiotics and microbiome-based interventions (Dubinsky et al., 2020).

We found that ARGs were resistant to antibiotics in Reserve Class. Reserve Class antibiotics were treated as the “last resort” options and usually used for highly selected patients (life-threatening infections due to multidrug-resistant bacteria). They were closely monitored and prioritized as targets of stewardship programs to ensure their continued effectiveness (World Health Organization, 2019). However, drug-resistance species in the CRC group were resistant to the “last resort” antibiotics, such as penems, glycylcyclines, and streptogramins. Increasing resistance of intestinal bacteria to “last resort” antibiotics reduces the number of antibiotics that can be used to control intestinal infections. Therefore, even the use of reserve group antibiotics still calls for caution.

Our study was limited to illustrating the association between CRC and antibiotic use for lacking information on antibiotic administration in these studies. A more directed study was needed to establish their association in the future. As our metagenomic data were collected from eight cohorts, the abundance of rSGBs and ARGs was affected by the host properties. Two cohorts were selected to analyze the abundance variation of rSGBs and ARGs, and consistent results were obtained. In our study, *E. coli* carried many MRGs and was significantly enriched in the CRC group. Although phylotype D *E. coli* (*E. coli D*) was one of these species with cyclomodulin-encoding genes and could produce cytotoxic necrotizing factors, phylotype B2 *E. coli* was the main strain associated with CRC (Wassenaar, 2018). Therefore, whether the phylotype D *E. coli* is associated with CRC warrants further investigation.

## Conclusion

We analyzed the species and ARGs distribution in the gut microbiota of CRC and CTR groups and found that the CRC group’s gut microbiota had higher ARGs and MRG abundance than that of the CTR group. And bacteria with ARGs were enriched in the CRC group, such as *E. coli*, *P. distasonis*, *B. thetaiotaomicron*, and *B. fragilis*. Meanwhile, CRC-associated species carried abundant ARGs. *E. coli* was the primary antibiotic resistance reservoir of species in the CRC patients. Using species and ARGs could classify CRC patients from healthy controls. It showed that the gut microbiota in CRC patients could confer resistance to fluoroquinolones, cephalosporins, penams, and tetracyclines. Our investigation proposes antibiotic resistance guidance to CRC patients, and this may help develop antibiotic use strategies to reduce the detrimental effects of antibiotic resistance.

## Materials and Methods

### Datasets and Samples Details

We downloaded a total of 769 metagenomic paired-end data, including 382 CRC patients (CRC group, aged 64 ± 11 years) and 387 healthy controls (CTR group, aged 61 ± 10 years) (Table 1). Data were selected from eight published studies with the NCBI SRA database accession codes PRJEB10878 (Yu et al., 2017), PRJNA389927 (Hannigan et al., 2018), PRJEB12449 (Vogtmann et al., 2016), PRJEB27928 (Wirbel et al., 2019), PRJEB6070 (Zeller et al., 2014), PRJNA447983 (Thomas et al., 2019), PRJEB7774 (Feng et al., 2015), and PRJDB4176 (Yachida et al., 2019). Participants who had a history of cancers, used antibiotics in the past period, or with gastrointestinal disease, including inflammatory bowel disease and intestinal infection, were excluded from the CTR groups (Zeller et al., 2014; Feng et al., 2015; Vogtmann et al., 2016; Yu et al., 2017; Hannigan et al., 2018; Thomas et al., 2019; Wirbel et al., 2019; Yachida et al., 2019). Basic information of participants, including gender, age, body mass index (BMI), vegetarian or not, smoking or not, health stat (health or CRC), and the American Joint Committee on Cancer Staging (AJCC Staging) information for CRC participation, was also collected (Supplementary Table 1).

### Metagenomes *de novo* Assembly, Binning, and Quality Evaluation

All the 769 paired-end fastq data went through quality control by fastp (Chen et al., 2018); the host sequence (human reference genome version: hg38) in the data was removed using soap2 (Li et al., 2009). Next, data were applied *de novo* assembled using metaSPAdes genome assembler (Nurk et al., 2017). Metagenomic binning was performed by MetaBAT2, which generated 36,461 bins in total. Completeness and contamination rates of bins were calculated by checkm qa workflow (Parks et al., 2015). We filtered bins into high-quality bins (completeness > 90%, contamination < 5%), medium-quality bins (completeness > 50%, contamination < 5%), and low-quality bins (the residual bins). To obtain more high-quality MAGs, we rebinned contigs using the same parameters mentioned previously for these contigs tagged “bin.unbinned” in the MetaBAT2 results and low-quality bins.

### Species-Level Genome Bin Cluster and Representation Selection

The completeness and contamination rates and the quality filtering of new bins were assessed again to remove low-completeness and high-contamination bins. Finally, we obtained 5,880 high-quality MAGs and 5,390 medium-quality MAGs. The 5,880 high-quality MAGs were clustered into species-level genome bins (SGBs) by a two-step clustering strategy based on genetic distance calculation by Metapi (Zhu et al., 2019). Then, representative genomes were selected for each cluster by SGB properties, including completeness, contamination, genome size, and strain heterogeneity index. The sequence with the maximal rank value was selected as representative genomes (rSGBs). The maximal rank value was computed according to a formula: *Rv* = *Cp* − *Ct* + log(*Gs*) − *Th*, where *Rv* means rank value, *Cp* and *Ct* represent completeness value and contamination value, and *Gs* and *Th* represent genome size and train heterogeneity.

### Taxonomy and Relative Abundance of Species

Taxonomy annotation for all the rSGBs was performed by GTDB-Tk (Chaumeil et al., 2019) based on the genome taxonomy database (GTDB, Release 95) (Parks et al., 2020). The high-quality reads were aligned to the rSGBs by bwa (default parameters) (Li, 2013). Sequence-based contigs abundance profiling was performed by jgi_summarize_bam_contig_depths (default parameters) (Kang et al., 2019). Reads were mapped to the rSGBs, and the number of reads counted formed a mapping depth. Considering the different sequencing depths of different samples, we used the mapping depth matrix of normalization to estimate the abundances of contigs. For the rSGB profile, we used the species assignment of each contig from the rSGBs and took the median of the relative abundance of contigs from the same rSGBs to generate the abundance of certain rSGBs (Supplementary Table 11). Then the α diversity (Shannon–Wiener index) of relative abundance of species was computed by the vegan (Oksanen et al., 2018). Next, β diversity was computed by PCoA and NMDS based on ape packages (Paradis and Schliep, 2018).

### Plasmid Assembly and Acquisition of Non-redundant Gene Catalog in the Plasmids

To avoid the possible ARG omission in the progress of MAGs assembly, we assembled the whole plasmid sequence in the gut microbiota from the host genome and removed high-quality reads using the metaplasmidSPAdes assembler (Antipov et al., 2019). Then, we called the genes in the contigs by MetaGeneMark (Zhu et al., 2010) and processed the gene cluster to the genes by cd-hit (Li and Godzik, 2006) to get the non-redundant gene catalog of the plasmids. After that, we computed the relative abundance of non-redundant genes in all samples. The genes were annotated to the NCBI-NT database (20191213) to get the species source of plasmids.

### Antibiotic Resistance Gene Identity, Resistance Mechanism, and Drug Type Analysis

To annotate ARGs in rSGBs, we predicted the open reading frame by Prodigal (Hyatt et al., 2010) and then identified ARGs using Resistance Gene Identifier based on CARD (version 3.0.7) for both rSGBs and plasmid genes (Alcock et al., 2020). Then, antibiotic resistance ontology (ARG types) was matched to the species by contigs id. Based on the best hit antibiotic resistance ontology results, relative abundances of ARGs, drug types, and resistance mechanism types were obtained. In our research, the ARGs were used to represent ARGs types. We also matched resistance antibiotics of rSGBs to AWaRe classification according to the WHO AWaRe classification of antibiotics (2019 version) (Supplementary Table 9). We computed the α diversity (Shannon–Wiener index) of ARG relative abundance in the rSGBs by the vegan (Oksanen et al., 2018). Then, PCoA and NMDS were performed to compute β diversity by ape packages (Paradis and Schliep, 2018). Meanwhile, PERMANOVA was used to test the statistical significance of β diversity by the vegan package (Oksanen et al., 2018).

### Machine Learning Train Model

To assess the classification effect of species and rSGB-sourced ARGs, we built and trained a series of machine learning models using selected elements from relative abundance profiles of species and ARGs. Data splitting, preprocessing, feature selection, model training, model tuning, and variable importance estimation were finished using the caret package (Kuhn, 2020). Before model training, near-zero variance and high correlation (absolute value of correlations coefficient > 0.75), variables were removed. And then, data were centered and scaled. Next, the 10-fold cross-validation approach was used to select features, and random forest methods were applied to train models. Finally, we assessed the effect of models by the area under the receiver operating characteristic curve (AUC) value using the ROCR package (Sing et al., 2005).

### Statistical Analysis

During the analysis, we carried out Wilcoxon test and LefSe analysis for the relative abundance of all species (Segata et al., 2011), with cutoff *p* < 0.05 and absolute values of the LDA score > 2.0 (Supplementary Table 6). Then, we analyzed the ARG profile by calculating Wilcoxon test with a cutoff of adjusted *p* < 0.05. Finally, 50 ARGs were filtered from rSGBs. To access the effect of ARGs in the CRC-associated species, we marked the selected 60 species above as “CRC-associated” cluster and other species with ARGs as “unassociated” cluster. And then, we compared the sum of ARG abundance in these two clusters, and then a PCoA analysis was performed on the classes. During the analysis and figure visualization, ggplot2 (Wickham, 2016), ggtree (Yu, 2020), ggpubr (Kassambara, 2020), and networkD3 (Allaire et al., 2017) packages were used in our study.

## Data Availability Statement

All the 5880 SGBs and 696 rSGBs data in this study have been deposited into CNGB Sequence Archive (CNSA) (Guo et al., 2020) of China National GeneBank DataBase (CNGBdb) (Chen et al., 2020) with accession number CNP0001862.

## Ethics Statement

The studies involving human participants were reviewed and approved by the Ethical Clearance the Institutional Review Board of BGI. Written informed consent for participation was not required for this study in accordance with the national legislation and the institutional requirements.

## Author Contributions

CL and ZL downloaded and analyzed the data. CL wrote the manuscript. JD modified the manuscript. HZ and CN gave beneficial advice. ZL and MF conceived the study and commented on the manuscript. All authors contributed to the article and approved the submitted version.

## Conflict of Interest

All authors were employed by company BGI-Shenzhen.

## Publisher’s Note

All claims expressed in this article are solely those of the authors and do not necessarily represent those of their affiliated organizations, or those of the publisher, the editors and the reviewers. Any product that may be evaluated in this article, or claim that may be made by its manufacturer, is not guaranteed or endorsed by the publisher.
