# Supplementary material for: Species-Level Analysis of the Human Gut Microbiome Shows Antibiotic Resistance Genes Associated With Colorectal Cancer
Source: Front Microbiol. 2021 Dec 15;12:765291. doi: 10.3389/fmicb.2021.765291 (PMC8715872; doi:10.3389/fmicb.2021.765291)
Supplement: Supplementary file 1 [file Data_Sheet_1.docx]

**Supplementary information for:**

**Species-level analysis of the human gut microbiome shows antibiotic resistance genes associated with colorectal cancer**

**Chuanfa Liu^1,2,3†^, Zhiming Li^2,3†^, Jiahong Ding^2,3†^, Hefu Zhen^2,3^, Mingyan Fang^2,3*^, Chao Nie^2,3*^**

^1^ College of Life Sciences, University of Chinese Academy of Sciences, Beijing 100049, China

^2^ BGI-Shenzhen, Shenzhen 518083, China

^3^ China National GeneBank, BGI-Shenzhen, Shenzhen 518120, China

**^†^** These authors are co-first authors of the study.

***Corresponding Authors:**

Chao Nie [niechao@genomics.cn](mailto:niechao@genomics.cn); Mingyan Fang [fangmingyan@genomics.cn](mailto:fangmingyan@genomics.cn)

**1. SUPPLEMENTARY FIGURES**

**
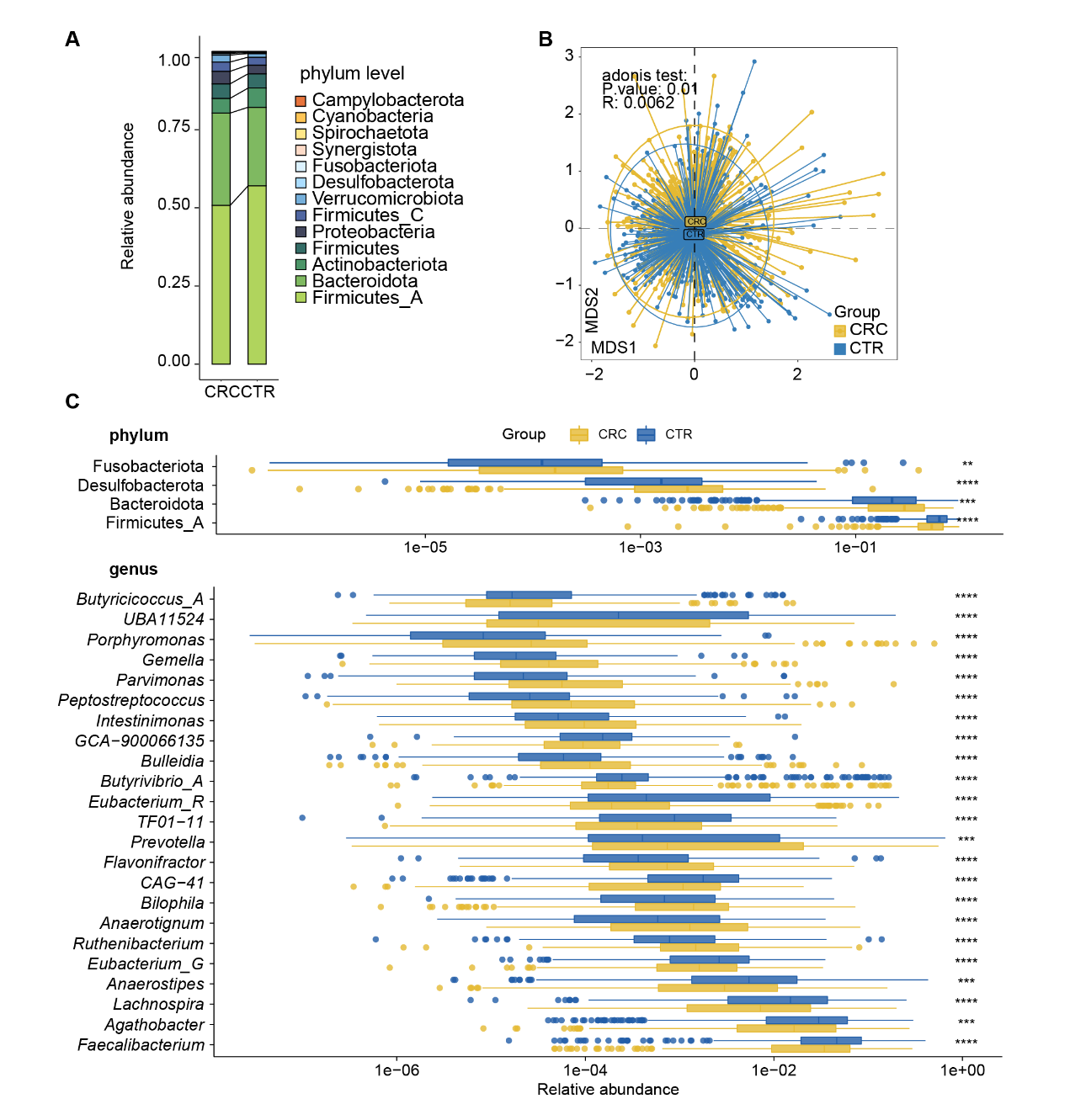
**

**Supplementary Figure S1.** **Abundance variation of species of CRC and CTR groups.** **A**. Relative abundance of species on the phylum level. **B**. NMDS analysis of species abundance. **C**. significant difference of species abundance on the genus (upper part) and phylum (lower part) levels.

**
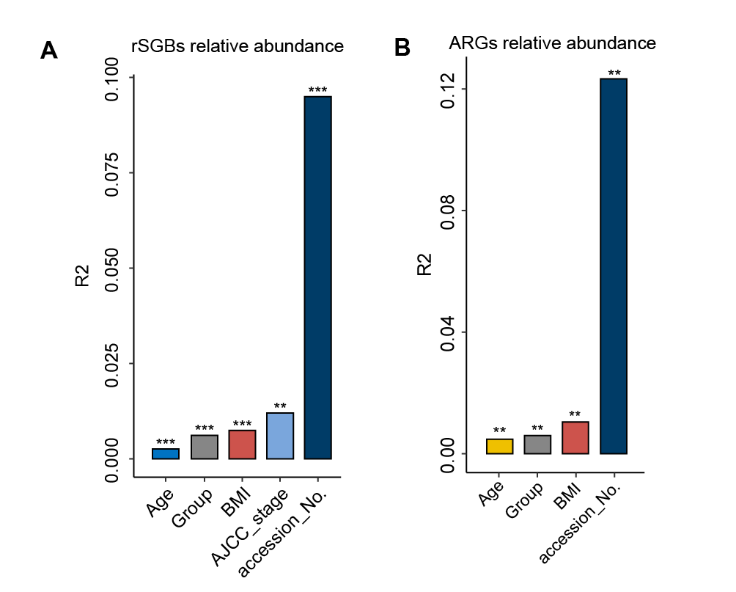
**

**Supplementary Figure S2. Impact of host properties on the gut microbiota. A**. the host properties impact on the rSGBs abundance **B.** the host properties impact on the ARGs abundance (In this figure, ** means *P*-value < = 0.01, *** means P-value <= 0.001).

**
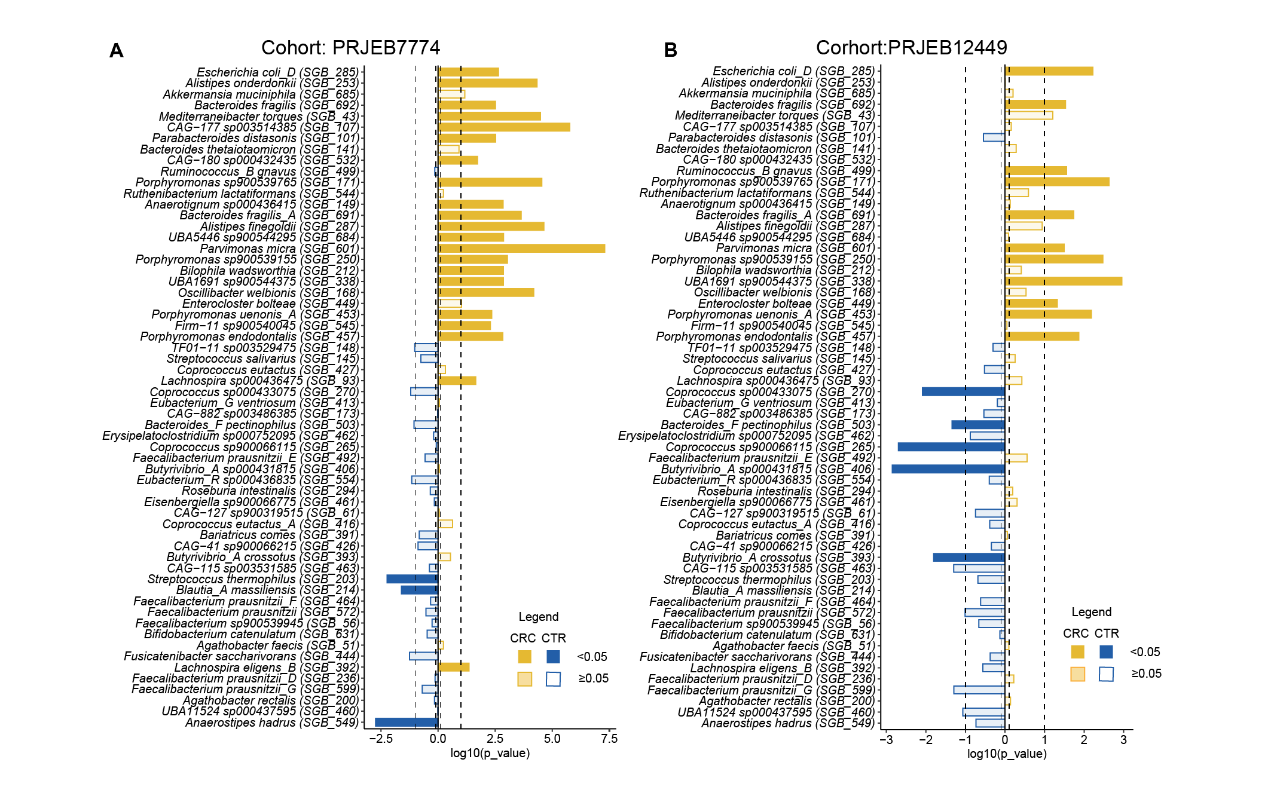
Supplementary Figure S3. Relative abundance difference of rSGBs in the CRC and CTR groups of two cohorts. A.** RSGBs abundance difference in the CRC (yellow color) and CTR (blue color) groups of PRJEB7774 cohort (sample count = 109). **B.** RSGBs abundance difference in the CRC (yellow color) and CTR (blue color) groups of PRJEB12449 cohort (sample count = 104). The deeper color in legends means *p*-value less than 0.05, and lighter color means *p*-value larger than or equal to 0.05.

**
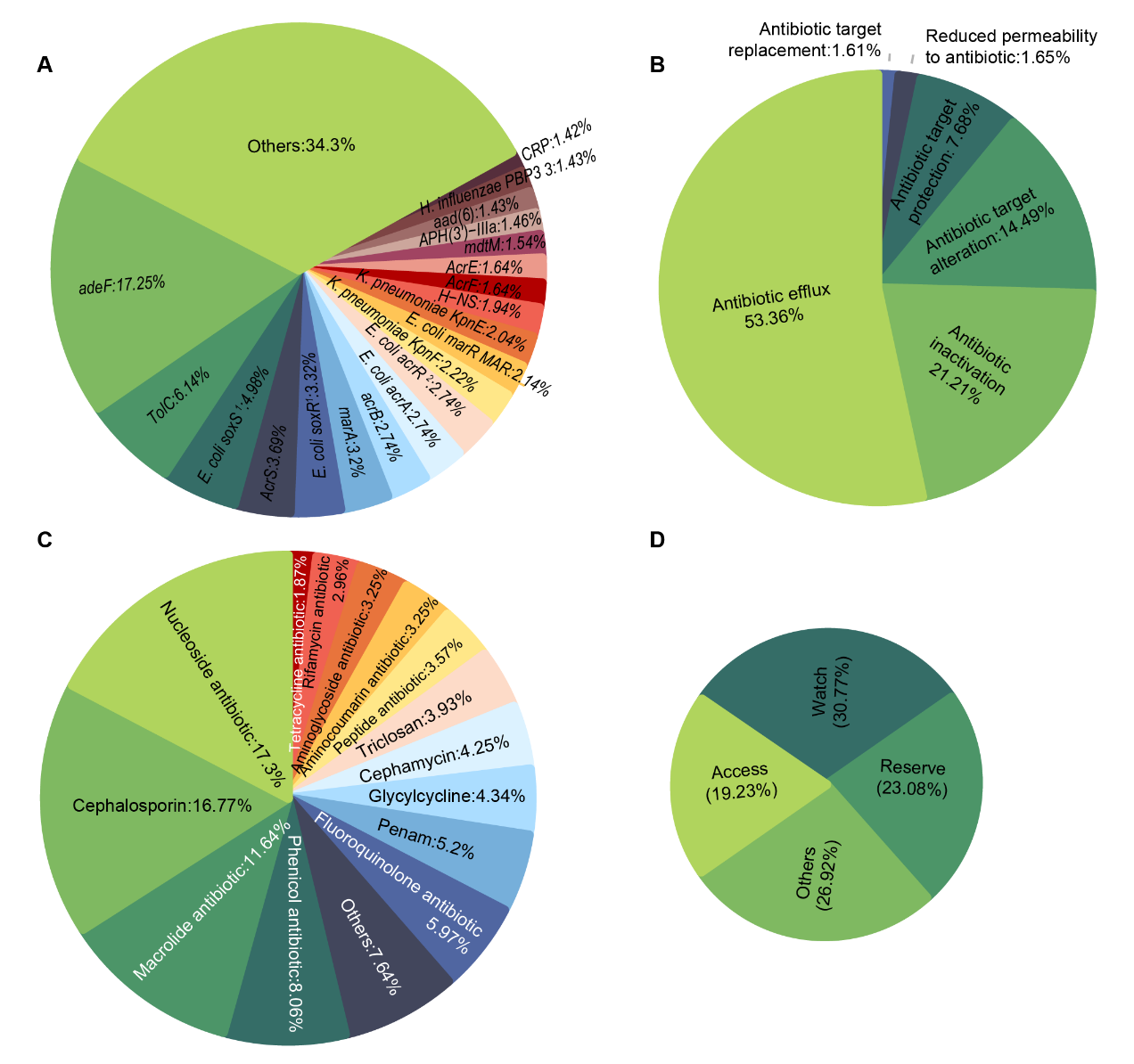
**

**Supplementary Figure S4. Resistance burden in the gut microbiota. A.** The abundance percentage of ARGs. **B.** The abundance percentage of ARGs resistance mechanism. **C.** The abundance percentage of drug resistance types. **D.** The percentage of resistance drugs in WHO AWaRe classification.

**
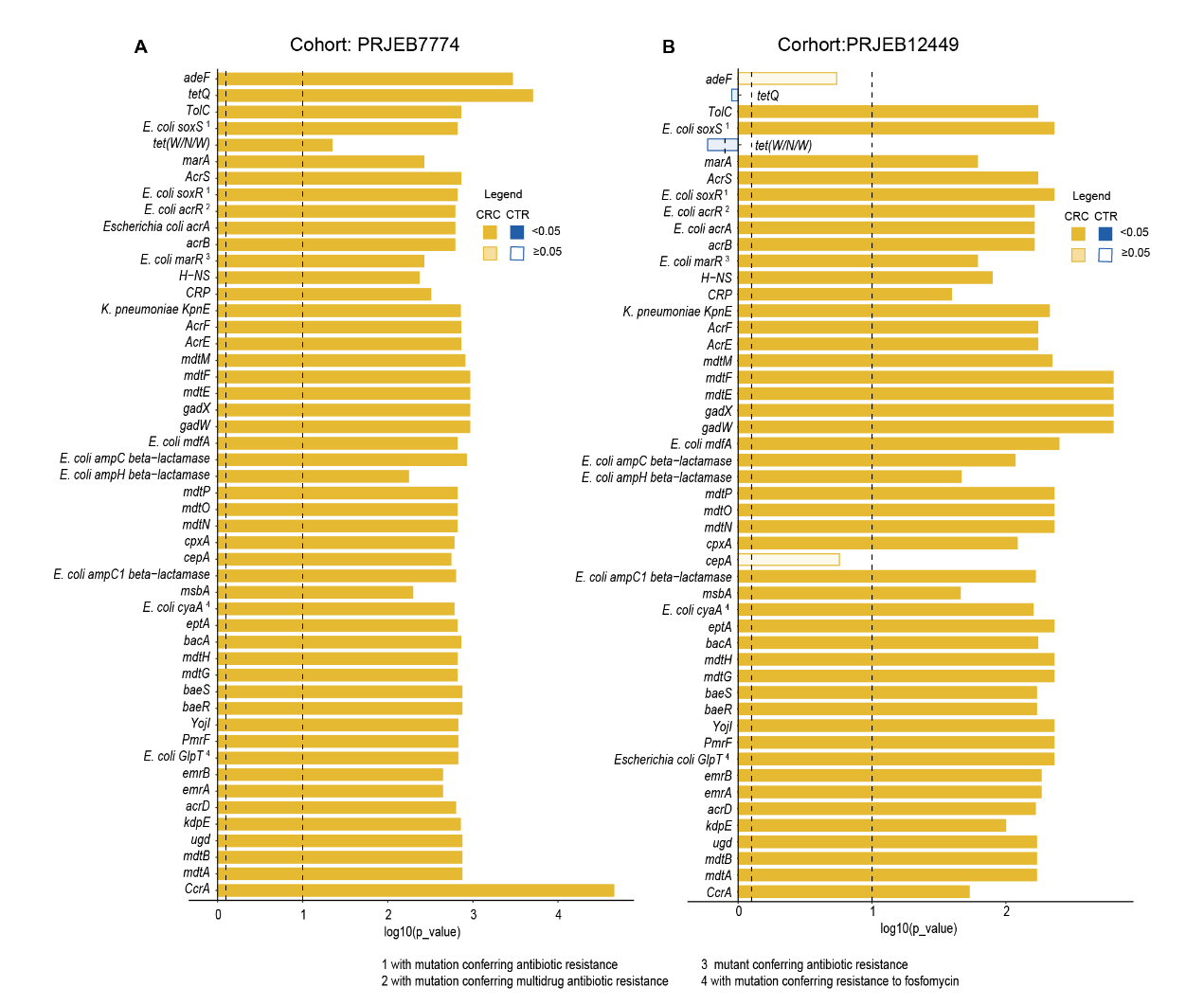
Supplementary Figure S5. The ARGs in the rSGBs were enriched in the CRC group of validation cohorts significantly. A.** ARGs in the PRJEB7774 cohort were significantly enriched in the CRC (yellow color) group, and a few ARGs were enriched in CTR (blue color) group. **B.** ARGs in the PRJEB12449 cohort were significantly enriched in the CRC (yellow color) group, and some ARGs were enriched in CTR (blue color) group. The deeper color in legends means *p*-value less than 0.05, and lighter color means *p*-value larger than or equal to 0.05.

**
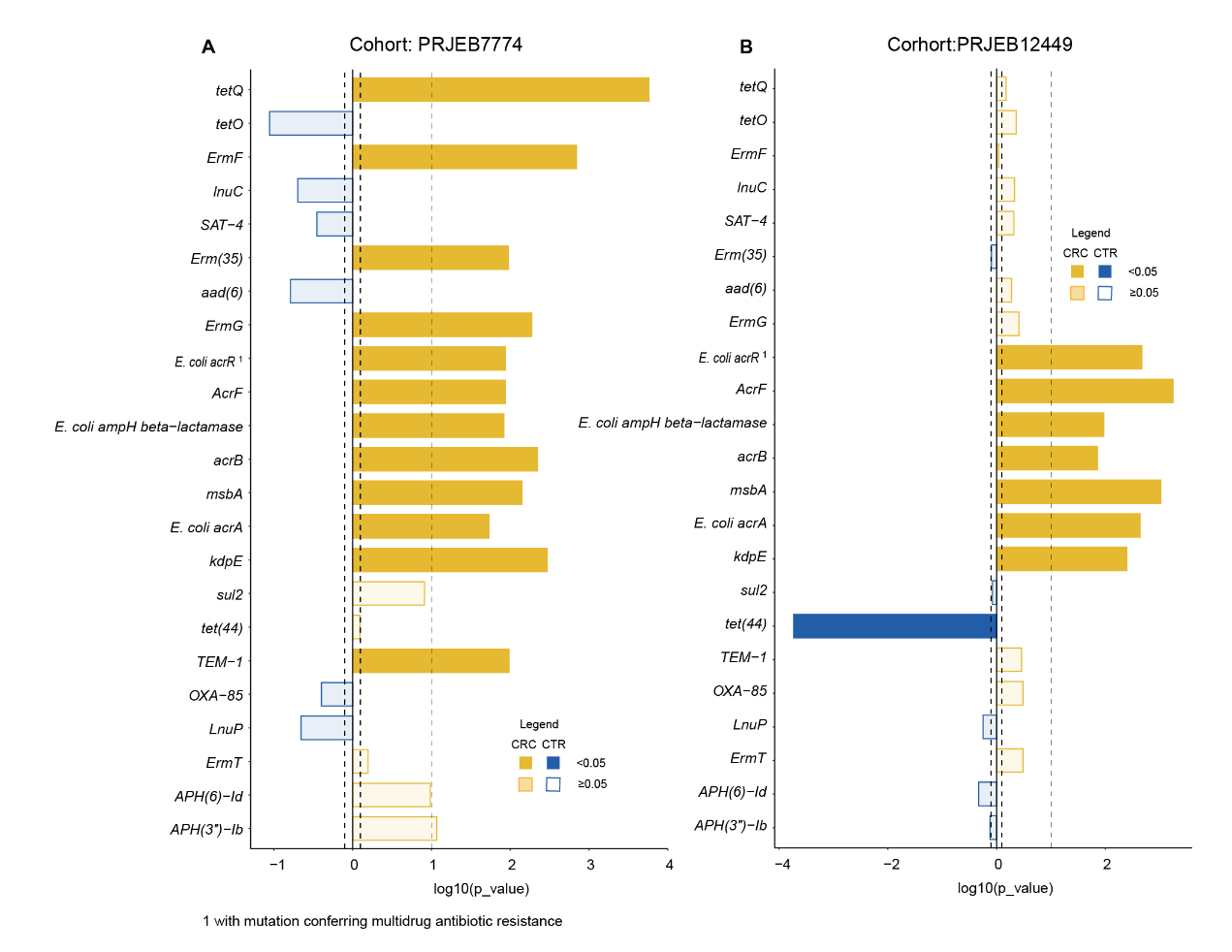
 Supplementary Figure S6. Relative abundance difference of ARGs in the plasmids of two cohorts. A.** the abundance difference of plasmids ARGs in the CRC (yellow color) and CTR (blue color) groups of PRJEB7774 cohort (sample count = 109). **B.** Plasmid ARGs abundance difference in the CRC (yellow color) and CTR (blue color) groups of PRJEB12449 cohort (sample count = 104). The deeper color in legends means *p*-value less than 0.05, and lighter color means *p*-value larger than or equal to 0.05.

**
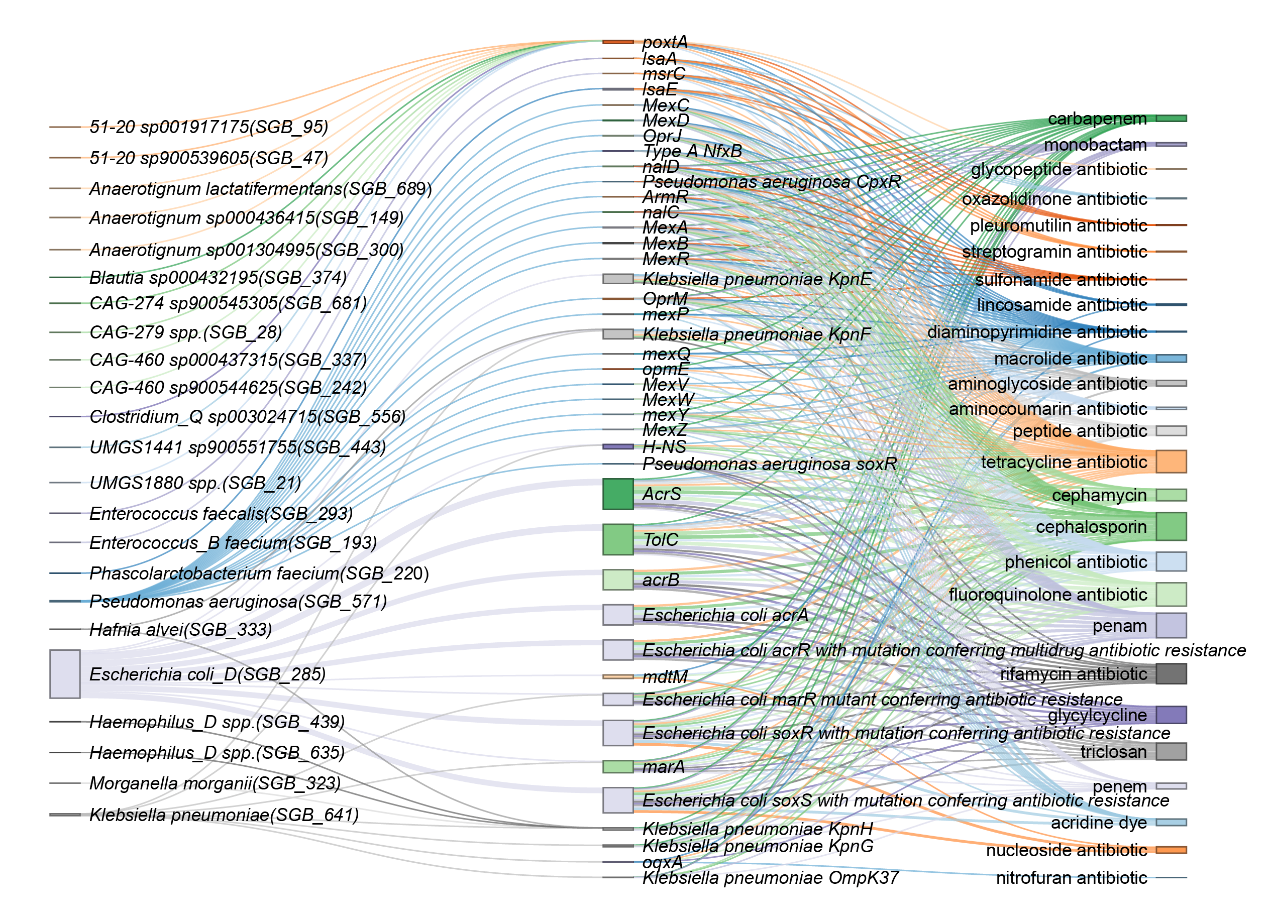
**

**Supplementary Figure S7. Resistance drug types and carriers of the identified MRGs.** The color of blocks and lines means different types, the width means the abundance percentage.


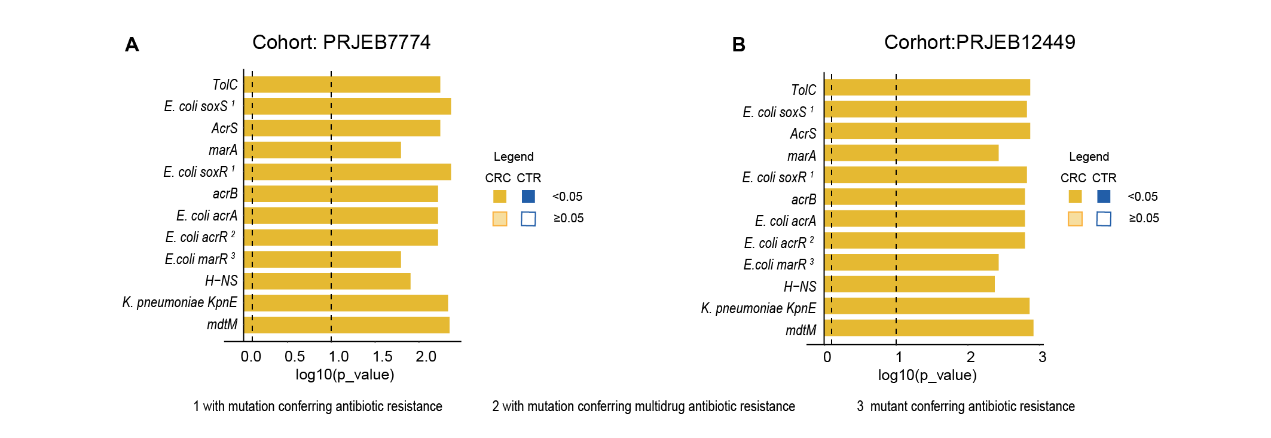
**Supplementary Figure S8. The MRGs were enriched in the CRC group of two cohorts significantly. A.** MRGs in the PRJEB7774 cohort were significantly enriched in the CRC (yellow color) group, and a few ARGs were enriched in CTR (blue color) group. **B.** MRGs in the PRJEB12449 cohort were significantly enriched in the CRC (yellow color) group, and some MRGs were enriched in CTR (blue color) group. The deeper color in legends means *p*-value less than 0.05, and lighter color means *p*-value larger than or equal to 0.05.

**
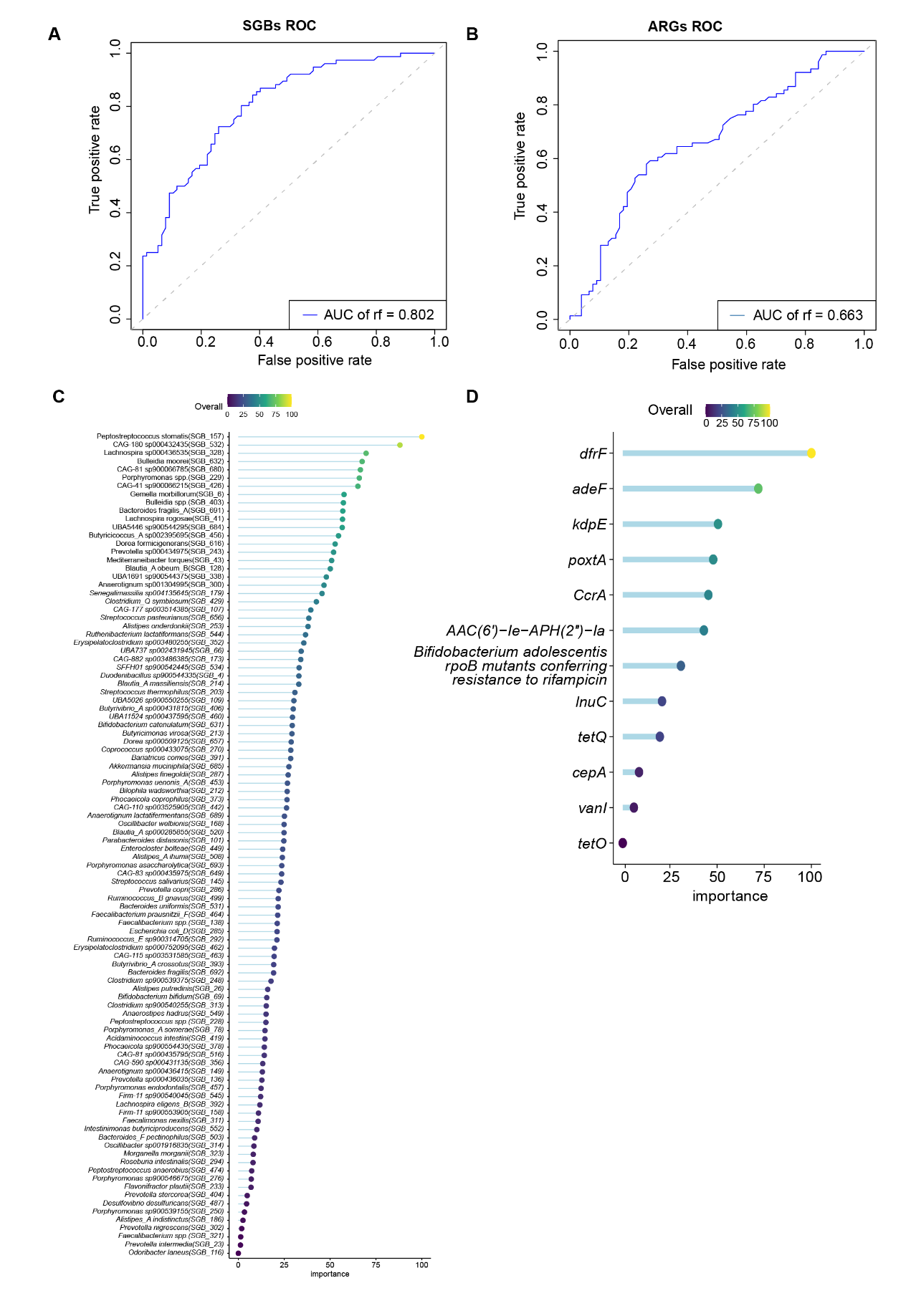
**

**Supplementary Figure S9. AUROC and features for the classification models. A.** AUROC of random forest model using all the species. **B.** AUROC of random forest model using all the ARGs. **C.** Selected species features importance used for random forest model. **D.** Selected ARGs features importance used for random forest model.

**2. SUPPLEMENTARY TABLES**

**Captions and introduction for the Supplementary tables:**

**Supplementary Tables:**

**Table S1. Sample information.** The letters M and F mean male and female in the gender column, respectively. The Roman numbers in the AJCC_stage column mean the different stages.

**Table S2. Information of 5880 high-quality MAGs.** In this table, genome size, GC ratio, completeness, contamination, and strain heterogeneity information of MAGs were recorded.

**Table S3. Data volume of samples.** The table recorded data volume, number, length, and the ratio of downloaded data, remove host sequence clean data, high-quality reads, assembled contigs, and SGBs in each sample.

**Table S4. 5880 MAGs cluster into 696 SGBs by mash and fastANI.** rSGBs was the representative of the genome_list, and rSGBs was marked with an SGB name.

**Table S5. Taxonomy details of rSGBs.** Taxonomy details, genome size, cultured or not, and reference genome in GTDB of rSGBs were recorded.

**Table S6. Selected rSGBs features from LefSe result**. This table recorded the average of rSGBs abundance in CRC and CTR groups, *P*-value and adjusted *P*-value of Wilcoxon test, and LDA score results.

**Table S7. ARGs in the rSGBs details.** In this table, ARGs annotation results were included, and ARGs' drug class and mechanism were recorded. For ARGs that could confer resistance to more than five types of antibiotics, we marked ‘yes’, otherwise, ‘no’.

**Table S8. ARGs details in the plasmid of gut microbiota.** In this table, annotation results of ARGs in the plasmid were included, and ARGs' drug class and mechanism were recorded. For ARGs that could confer resistance to more than five types of antibiotics, we marked ‘yes’, otherwise, ‘no’.

**Table S9. Resistance drug class and WHO AWaRe classification of antibiotics**. **ACCESS**: first or second choice antibiotics offer the best therapeutic value while minimizing the potential for resistance; **WATCH**: first or second choice antibiotics, only indicated for a specific, limited number of infective syndromes, more prone to be a target of antibiotic resistance and thus prioritized as targets of stewardship programs and monitoring; **RESERVE**: ‘last resort’, highly selected patients (life-threatening infections due to multi-drug resistant bacteria), closely monitored and prioritized as targets of stewardship programs to ensure their continued effectiveness.

**Table S10. Species and ARG term features were used in the classification model.** The upper part was the importance of species, and the lower part was the importance of ARGs.
